# The antihyperlipidemic drug potassium piperonate impairs the migration and tumorigenesis of breast cancer cells *via* the upregulation of miR-31

**DOI:** 10.3389/fonc.2022.828160

**Published:** 2022-10-13

**Authors:** Xiaoxia Tian, Junping Lu, Kathleen Nanding, Linzhe Zhang, Yanrong Liu, Mailisu Mailisu, Morigen Morigen, Lifei Fan

**Affiliations:** State Key Laboratory of Reproductive Regulation and Breeding of Grassland Livestock, School of Life Sciences, Inner Mongolia University, Hohhot, China

**Keywords:** potassium piperate, miR-31, breast cancer, cell migration, drug combination

## Abstract

**Background:**

Breast cancer is the second cause of cancer death in women, and tumor metastasis is the primary cause of mortality. Due to the involvement of many regulatory molecules and signaling pathways, the occurrence and development of metastases needs to be further studied. MicroRNAs (miRNAs) are ubiquitously expressed small non-coding RNAs that have been shown to play an important role in the diagnosis and treatment of many diseases, as well as representing an attractive candidate for metastasis control. In this study, we investigated the mechanism of potassium piperonate (GBK) in impairing breast cancer cell invasion and metastasis by targeting miR-31.

**Methods:**

Breast cancer cells, either treated with GBK or left untreated, were assessed for migration and invasion capacities using wound healing and transwell assays. GBK-targeted miRNAs were identified and verified using RT-qPCR. Western blotting was used to validate the changes in expression levels of miR-31-targeted genes. Methylation specific PCR was performed to detect the effect of GBK on the methylation levels of the lncRNA *LOC554202* host gene. The synergistic effect of GBK and the chemotherapy drug cisplatin (DDP) on breast cancer cells was verified using cell proliferation, colony formation, and RT-qPCR assays *in vitro*, and the tumor xenograft model *in vivo.*

**Results:**

We found that miR-31 was the main target of GBK. GBK treatment affected the epigenetic modification at CpG sites by downregulating DNA methyltransferases. Thus, the CpG-associated methylation levels of lncRNA *LOC554202* decreased significantly, and in turn upregulated both miR-31 and its host gene *LOC554202* in breast cancer cells. We also observed the significant inhibition of miR-31-targeted genes following GBK treatment, including *RHOA*, *WAVE3*, and *SATB2*, with functions closely related to cancer cell invasion, migration, and proliferation. Furthermore, we revealed that the combination of GBK and DDP had a synergistic effect on inhibiting the proliferation of breast cancer cells *in vitro* and *in vivo*, especially in triple negative breast cancer (TNBC).

**Conclusions:**

This study investigated the target of GBK in the inhibition of breast cancer migration and invasion, and the underlying mechanisms involved, providing theoretical support for the development of GBK as an auxiliary drug for clinical treatment.

## Background

Breast cancer is now the most frequently diagnosed cancer and the second leading cause of cancer-related deaths in women worldwide (1). Strategies targeting the primary tumor have markedly improved, including surgery, chemotherapy, radiation therapy, hormone therapy, and targeted therapy. However, metastasis remains the greatest clinical challenge in breast cancer. The mechanisms implicated in tumor metastasis need to be further investigated in order to improve the long-term control of breast cancer progression. Previous research has shown that the deregulated expression of miRNAs is intimately associated with breast tumor invasion and metastasis (2).

In recent years, miRNAs have emerged as key players in the processes of gene expression regulation. Ubiquitously expressed miRNAs are approximately 19-24 nucleotides in length, and function by binding to the complementary sequences of their target mRNAs, leading to mRNA degradation as well as the subsequent downregulation or suppression of protein synthesis. miRNAs play a pivotal role in various cellular processes *in vivo*, such as proliferation, migration, cell death, and cell cycle regulation (3). The miRNA expression patterns differ among different subtypes of breast cancer: the let-7c, miR-10a, and let-7f miRNAs are associated with luminal type A breast cancer; miR-18a, miR-135b, miR-93, and miR-155 have been shown to be related to the basal cell subtype; while miR-142-3p and miR-150 have been shown to be associated with the HER2-positive subtype (4); and miR-10b, miR-26a, and miR-153 have been used as potential biomarkers for triple negative breast cancer (TNBC) 5). About 70% of breast cancers are estrogen receptor (ER) or progesterone receptor (PgR) positive. ER controls the expression of multiple genes and proteins through genomic and non-genomic pathways, whereas PgR is induced by ER, and PgR-related signal transduction pathways are closely related to the occurrence and development of breast cancer (4). The tumor suppressor miRNAs that have been identified in breast cancer include miR-206, miR-17-5p, miR-125a, miR-125b, miR-200, let-7, miR-34, and miR-31. On the contrary, the expression of miR-21, miR-155, miR-10b, miR-373, and miR-520c are positively correlated with the occurrence of breast cancer (6).

*Piper longum L.* (also called long pepper) is a plant used in traditional Chinese medicine, as a source of the antihyperlipidemic agents piperine, piperlonguminine, and pipernonaline (7). Potassium piperonate (GBK) is a derivative of piperine. GBK has the functions of reducing blood lipid levels and cholesterol, with efficacy comparable to that of the commercial antihyperlipidemic drug statins (8). In addition, previous studies have shown that GBK exerts an anti-tumor effect, especially in breast cancer. GBK can specifically inhibit the viability of a variety of breast cancer cells by arresting the cell cycle in G1 phase and inhibiting cell proliferation. Furthermore, GBK can induce breast cancer cell apoptosis through the mitochondria-dependent pathway. However, the potential of GBK to inhibit breast cancer cell invasion and metastasis has not been previously investigated.

In this study, we set out to uncover the mechanisms employed by GBK to impair breast cancer cell invasion and metastasis, and aimed to identify the main target of GBK by analyzing miRNA expression pattern changes after GBK treatment. Furthermore, we explored whether the growth and migration of breast cancer cells could be more efficiently inhibited when chemotherapy drugs were used in combination with GBK *in vitro* and *in vivo*. In summary, this study further assessed the underlying mechanisms involved in the GBK-mediated inhibition of breast cancer progression. Our finding provides theoretical support for the development of GBK as an auxiliary drug for the clinical treatment of breast cancer.

## Methods

### Cell strains and cell culture

MCF-10A, MCF-7 and SUM-159 were purchased from National Infrastructure of Cell Line Resource of China.

MCF-10A cells were maintained in F12 medium (Gibco) supplemented with 5% horse serum (Gibco), 1%(vol/vol) penicillin/streptomycin/L-Glutamin (Gibco), 10 mg/mL insulin, 20 mg/mL EGF, 100 mg/mL cholera toxin and 0.5 mg/mL hydrocortisone. SUM-159 cells were maintained in F12 medium (Gibco) supplemented with 5% fetal bovine serum (GEMINI), 1% (vol/vol) penicillin/streptomycin/L-Glutamin (Gibco), 5 mg/mL insulin and 10 mg/mL dexamethasone. MCF-7 cells were maintained in Dulbecco’s Modified Eagle Medium (Gibco) supplemented with 10% (vol/vol) fetal bovine serum (GEMINI) and 1% (vol/vol) penicillin/streptomycin/L-Glutamin (Gibco). All cells were cultured at 37°C, 5% CO_2_ in a humidified atmosphere.

### Reagents and drugs

GBK is a generous gift from Professor Gereltu Borjihan of Inner Mongolia University. The purity of Piperine is 99% detected by high pressure liquid chromatography (**Supplementary Figure 1**). miRNeasy^®^ Serum/Plasma Kit (Qiagen, USA); TransZol Up Kit (TransGene Biotechnology Co., Ltd.); Mir-XTM miRNA First-Strand Synthesis Kit (TaKara); TransScript One-Step gDNA Removal and cDNA Synthesis SuperMix Kit (TransGene Biotechnology Co., Ltd.); SYBR^®^ Premix Ex TaqTM II Kit (TaKara); CCK-8 Kit (Beyotime Biotechnology Co., Ltd.); Crystal Violet (SIGMA); FITC Annexin V Apoptosis Detection Kit I (BD Pharmingen); Protein Antibody (Absin); Tubulin Antibody (TransGene Biotechnology Co., Ltd.); PierceTM ECL Western Blotting Substrate Kit (Thermo, USA); ELISA Kit (Wuhan Xinqidi Biotechnology Co., Ltd.); TransStart FastPfu DNA Polymerase (TransGene Biotechnology Co., Ltd., Lot: M10524); Cisplatin (DDP, Meilun Bio, Lot: D0921A); Etoposide (VP-16), United Laboratories).

### Primers

The primers used in the real-time quantitative PCR (RT-qPCR) or PCR were designed by Primer 5 software and NCBI Primer-BLAST, synthesized by Shanghai Biotech (Sangon) Beijing Primer Synthesis Company, and Tm value fluctuated at 60°C. Specific primers are shown in **Supplementary Tables 1–3**.

### miR-31 mimics and inhibitor

miR-31 mimics and inhibitor were synthesized by Biomics biotechnologies (China, Jiangsu) and the sequences are shown in **Supplementary Table 4**.

### Western blot

Cells were seeded in 6-well plates. The cells were harvested 2 days after GBK added, washed once in PBS, and lysed in sample buffer (2% SDS, 0.25 M pH 6.8 Tris-HCl, 20 mM dithiothreitol, 10% glycerol, 0.1% Bromophenol blue). 20 μg of protein was separated on 12% Polyacrylamide gel and transferred to a nitrocellulose membrane. Membrane was blocked for 1 hour at room temperature in blocking buffer (5% skim milk in PBS containing 0.05%Tween-20) and then incubated with primary antibodies and peroxidase (HRP)-conjugated secondary antibody. Tubulin or GAPDH was served as a reference protein.

### Cell proliferation ability assay with CCK-8

5×10^3^/100 μL cells were seeded in a 96-well plate and incubated at 37°C, 4% CO_2_. After 24 hours, the cells were treated with GBK. After 48 hours, 10 μL CCK-8 solution was added into each well and returned to the incubator for further 1.5 hours. The viability of the cells was measured using a microplate reader at the wavelength of OD_450_nm.

### Extraction of miRNA from mouse serum samples

The NOD/SCID mice used in this experiment were purchased from Boai Biotechnology Co., Ltd. 12 female NOD/SCID mice aged 3-4 weeks were used to establish heterogeneous tumor models with MCF-7 breast cancer cells. Five mice were selected as the control group, and the other seven as the experimental group.

After the tumor volume reached 8 mm^3^, the experimental group was injected with GBK 10 mg/(kg.1D), while the control group was injected with 0.9% normal saline. Serum samples were taken 21 days after treatment.

Extraction of miRNA from mouse serum samples was performed exactly according to miRNeasy^®^ Serum/Plasma Kit (Qiagen, USA) instructions.

200 μL of serum sample were taken from each mouse, diluted in 5x volume of lysate, and after incubated for 5 mins at room temperature, 3.5 μL of the “control” solution was added. Next, 200 μL of chloroform was added and incubated at room temperature for 3 min. The clear supernatant was then transferred to a new EP tube, mixed with 1.5x volume of absolute ethanol and vortexed to mix. All the solution was added to the adsorption column, centrifuged at room temperature, 12000 rpm for 1 min. 700 μL of RWT buffer was added to the adsorption column, centrifuged. 500 μL of RPE buffer was added to the adsorption column, centrifuged. 500 μL of 80% ethanol was added to the adsorption column, centrifuged. The adsorption column was placed on a new collection tube and centrifuged. 14 μL of RNase-free water was added to the adsorption column and incubated for 1 min, then centrifuged. The extracted RNA was stored at -80°C until use.

### Relative quantitative real-time PCR

Extraction of total RNA from cell lines was carried out according to the TransZol Up Kit instruction. Reverse transcription PCR was performed using TransScript One-Step gDNA Removal and cDNA Synthesis SuperMix Kit (TransGene Biotechnology Co., Ltd.), and quantitative real-time PCR was performed using SYBR^®^ Premix Ex TaqTM II Kit (TaKara). GAPDH gene was served as a reference. Gene expression was measured in triplicates.

### Wound healing assay

Cells were seeded into 6-well tissue culture plate. When the cell density reached 100%, scratching and photographing were performed. Replaced old medium with the 1% low-serum medium and continue to culture cells in incubator after drugs were added. Photos were taken every 12 hours on an inverted microscope. The gap distance can be evaluated using Image J software to calculate the cell migration rate.

### Cell invasion experiment

The subpackaged matrix glue (Corning, US) was removed from -20°C and quickly diluted ten times with serum-free medium on ice. 100 μL diluted matrix glue was applied to the upper and lower surfaces of the transwell chamber in a 24-well plate, and then incubated at 37°C for 1 hour. 5 × 10^5^ cells/mL suspension was prepared and 200 μL was added to each chamber. Simultaneously, 600 μL medium with 5% FBS was added into the lower layer of the transwell chamber. The bottom of the upper chamber was checked for absence of bubbles and the whole plate was incubated at 37°C for 24 hours. A cotton swab was used to gently wipe the upper chamber and carefully remove the sidewall cells. 600 μL 4% PFA was added into each lower chamber and the cells fixed at room temperature for 20 mins. After the PFA was washed off using PBS, 600 μL 0.1% crystal violet was added into the bottom chamber, and the cells stained in the dark for 30 mins at room temperature. The chamber was then taken out and washed with PBS, and the number of cells transferred to the lower surface of the chamber was counted under the microscope.

### Cell colony formation assay

1×10^3^ cells were seeded into 6-well plate. After being cultured for 24 hours in an incubator, potassium piperonate (GBK), cisplatin (DDP) and etoposide (VP-16) were added with a concentration gradient for further 7-14 days. Then, 1 mL 4% PFA was added to each well for 15 minutes, cells were rinsed with 1 ml PBS, and 500 μL 0.01% crystal violet was added to each well for 15 minutes. The plates were dried and the colonies were counted.

### Methylation specific PCR (MSP)

Preparation of bisulfite-modified DNA for methylation analysis was performed according to the EZ DNA Methylation-Gold™ kit (Zymo Research) instructions. In brief, the CT Conversion Reagent and M-Wash Buffer were prepared prior to use. Genomic DNA (200 ng in 20 μL) was converted using 130 μL of CT Conversion Reagent in a PCR cycler, with the following cycle program: 98°C for 10 mins, 64°C for 2.5 hours, 4°C storage up to 20 hours. 600 μL of M-Binding Buffer was added, followed by a single washing step with M-Wash Buffer. 200 μL M-Desulphonation Buffer was added, and then incubated at RT for 20 mins and washed twice with M-Wash Buffer. 10 μL of double-distilled water was added, the samples was centrifuged, and then the DNA was resuspended.

MSP was performed on bisufite-converted DNA using the special primer pairs described in **Supplementary Table 3**. Every genomic DNA sample was amplified using either the unmethylated or the methylated primer pairs. The PCR products were next separated by agarose electrophoresis according to their densities, which corresponded to the intensities of the PCR products between the methylated and unmethylated primer-pairs.

### NOD/SCID mouse tumor xenograft model

The mouse model was derived from Jennio Biotechnology company. Twenty immunodeficient female mice between 3 and 4 weeks were used to establish a NOD/SCID mouse xenograft model. 100 μl of prepared 2×10^6^/100 μL MCF-7 cells suspension was injected into the hind leg of NOD/SCID mice, 4 nude mice were selected randomly as the control group, and the other 12 nude mice were divided into 3 experimental groups randomly. The body weight of mice was measured every 3 days after injection. When the tumor mass grew to 8 mm^3^, the drugs or 0.9% saline was injected into nude mice. Weight and the tumor volume measured with a vernier caliper were recorded every 3 days. The tumor volume was calculated according to the following formula: V=(ab^2^)/2 (“a” represents the longest diameter of the tumor and “b” represents the shortest). After 20 days, the tumor-bearing mice were killed, the tumor lumps were removed and weighed.

## Results

### GBK impairs the migration and invasion of breast cancer cells

In order to investigate the effect of GBK on the migration and invasion of breast cancer cells, would healing and transwell cell invasion assays were performed. The TNBC cell line SUM-159, the ER-positive breast cancer cell line MCF-7, and the normal human epithelial mammary cell line MCF-10A were scratched and photographed, prior to treatment with different concentrations of GBK. After 24 hours of culture, cell images were captured, and the cell motility changes were analyzed. In order to rule out the influence of cell proliferation, we switched to 1% low serum medium after the scratch treatment and took pictures within one cell cycle.

**Figure 1:**
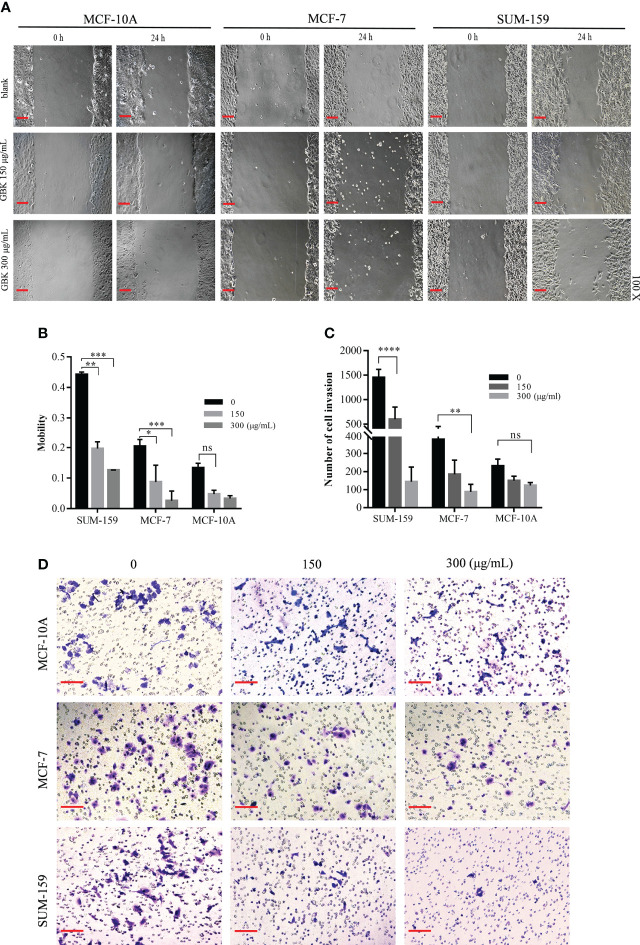
GBK impairs the migration and invasion ability of breast cancer cells **(A)** MCF-10A, MCF-7, and SUM-159 cells were scratched and treated with different concentrations of GBK. Cell images were captured after 24 hours (original magnification, ×10, scale bar (black) as shown). **(B)** The cell mobility was analyzed using Image J software. Cell motility = (t_0 h_ scratch width - t_24 h_ scratch width)/t_0 h_ scratch width. **(C)** Cell invasion after GBK treatment was investigated using the Matrigel invasion assay. Cell numbers were calculated by imaging five different fields, and the quantification of results is showed. **(D)** Photos of representative fields of cell invasion are shown. Data are presented as mean ± s.e.m. of at least three independent experiments. Student’s t test, *p < 0.05, **p < 0.01, ***p < 0.001, ****p < 0.0001. ns, no significance. Scale bar represents 20 μm.

The results showed that the wound closure ability of both SUM-159 and MCF-7 cells was significantly inhibited by GBK (**Figures 1A, B**). We observed a 59% reduction in cell migration in the 150 μg/mL (IC_50_ of SUM-159 cells) GBK treatment group, which further increased to 71% in the 300 μg/mL GBK treatment group (**Figure 1B**). Moreover, for the SUM-159 cells, GBK treatment inhibited invasion (in the transwell invasion assay) by 64% and 92% after treatment with 150 μg/mL or 300 μg/mL GBK, respectively (**Figures 1C, D**). The administration of GBK to the MCF-7 human non-invasive breast cancer cells also impaired cell invasion, but to a lesser extent (**Figures 1C, D**). GBK treatment had no significant influence on the migration and invasion of normal human epithelial mammary cell line MCF-10A (**Figures 1A–D**). Taken together, these data indicate the anti-migration and anti-invasion roles of GBK in breast cancer, especially in invasive breast cancer cells.

### Tumor suppressor miR-31 is upregulated after GBK treatment in breast cancer cells

miRNAs have been shown to function as tumor suppressors, implicated directly in the inhibition of cancer progression. Consequently, certain miRNAs may act as specific drug targets in cancer treatment. For instance, miR-21 is involved in the regulation of apoptosis in breast cancer cells (9), miR-151 is found to affect the development of breast cancer by modulation of DNA repair processes, and miR-421 can inhibit the migration and invasion of breast cancer by targeting *MTA1* (10, 11). On the basis of such research, we selected nine miRNAs closely associated with the occurrence and development of breast cancer. Of the nine miRNAs selected, miR-22, miR-31, miR-41, and miR-421 function as tumor suppressors, while miR-21, miR-145, miR-150, miR-182, and miR-217 have been shown to promote cancer development (10, 12–21). The downregulation of the tumor suppressor miR-22 in metastatic cancer cells has been shown to be associated with a disproportionately poor prognosis (16, 17). miR-31 inhibits the cell cycle by suppressing the expression of multiple factors involved in the regulation of DNA replication and cell cycle progression. In addition, increasing miR-31 or miR-421 levels have been shown to significantly inhibit the migration and invasion of MDA-MB-231 and MCF-7 cells (10, 15, 22). miR−411 downregulation in breast cancer is associated with lymph node metastasis and histological grade (22). miR-21 overexpression facilitates breast cancer cell proliferation and metastasis *in vivo*, and plasma miR-21 level is an important biomarker for breast cancer diagnosis 13). miR-145 reduces breast cancer cell migration and inhibits epithelial-mesenchymal transition (14). The upregulation of miR-150 in breast cancer is inversely associated with P2X7 receptor expression levels, which regulates cell growth through apoptosis (12, 23). In TNBC, miR-182 promotes cell proliferation and metastasis by targeting *FOXF2*, while miR-217 inhibits cell growth, migration, and invasion by targeting *KLF5* (18, 20). These miRNAs mainly affect the proliferation, migration, and invasion of breast cancer cells during the development of breast cancer.

**Figure 2:**
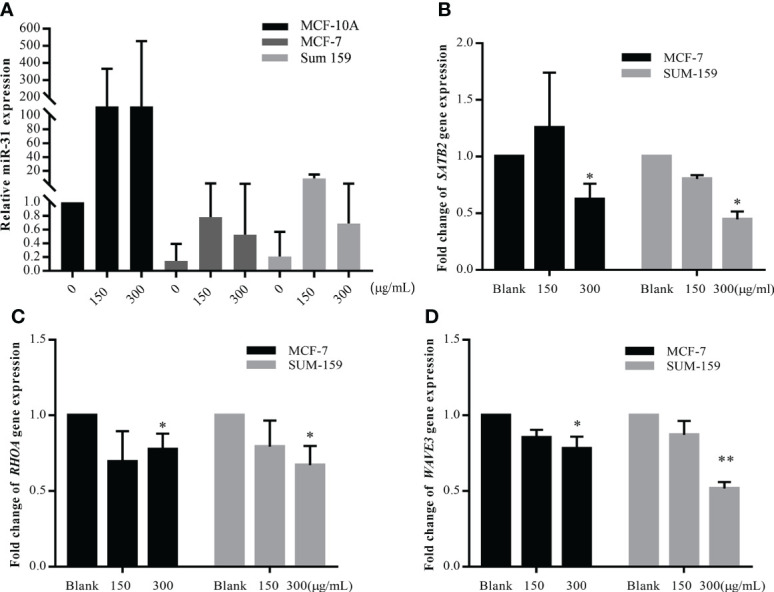
Detection of miR-31 and its target gene expression under the treatment of GBK in different breast cell lines. **(A)** MCF-10A, MCF-7, and SUM-159 cells were treated with different concentration of GBK. Total RNA was extracted after 48 hours for miRNA expression detection by RT-qPCR. Quantification of miR-31 expression in three different breast cell lines after application of different concentrations of GBK was shown. **(B-D)** Expression of *SATB2*, *RHOA* and *WAVE3* in MCF-7 and SUM-159 cells after treatment with different concentrations of GBK was analyzed by RT-qPCR. Data are presented as mean ± s.e.m. of three independent experiments. Student’s t test, *p < 0.05, **p < 0.01.

GBK can specifically inhibit the proliferation and migration of breast cancer cells. Therefore, we wanted to test the effect of GBK on the nine miRNAs described above. Initially, in order to determine the potential miRNA target of GBK, RT-qPCR was used to detect miRNA expression in serum samples derived from the breast cancer xenograft model mice after GBK treatment (**Supplementary Figure 2**). We found that the differential expression of miRNAs in serum were not significant in GBK-treated mice compared to that in the control group. We then investigated the effect of GBK treatment on the expression of candidate miRNAs in the breast cancer cell lines MCF-7 and SUM-159. Different concentrations of GBK were added to the cells for 48 hours and miRNA expression was detection by RT-qPCR. The expression of miR-31 was consistently upregulated after GBK treatment in the two breast cancer cell lines tested (**Figure 2A**). The differential expression pattern of other miRNAs were not consistent after GBK treatment and thus were excluded from further investigation (**Supplementary Figure 3**).

By analyzing the expression of nine breast cancer related miRNAs, our preliminary results indicated that the tumor suppression effect of GBK may be closely related to miR-31.

### The expression of miR-31 target genes related to cell migration is downregulated after GBK treatment

In previous studies, the function of miR-31 has been shown to be highly associated with the progression and metastasis of breast cancer. miR-31 plays a fundamental role in the regulation of the invasion-metastasis cascade by targeting critical genes, such as those involved in cytoskeletal rearrangement of cancer-associated fibroblasts (CAFs). *RHOA*, which participates in the regulation of the actin cytoskeleton, was shown to be a direct target of miR-31 (24, 25). Moreover, another member of the Rho family, *RHOBTB1*, was shown to be a target of miR-31 in colon cancer (26). WAVE3, an actin remodeling protein, was shown to be overexpressed in invasive breast cancer cells due to miR-31 downregulation, and its expression promoted cancer cell migration and invasion (27). The homeobox gene *SATB2* was shown to be a direct target of miR-31 in CAFs and is involved in promoting tumor cell migration and invasion (4).

We screened nine target genes closely related to the anti-metastatic function of miR-31, monitoring their expression in MCF-7 and SUM-159 cells after GBK treatment. The expression levels of *RHOA*, *SATB2*, and *WAVE3* were all downregulated after 48 hours of GBK treatment, indicating that the tumor suppression effect exerted by GBK may be related to invasion/metastasis-associated signaling pathways (**Figures 2B–D**, **Supplementary Figure 4**). *UBC13*, which has a regulatory role in cell death (28), was also downregulated, although its function in tumor invasion is poorly understood.

**Figure 3:**
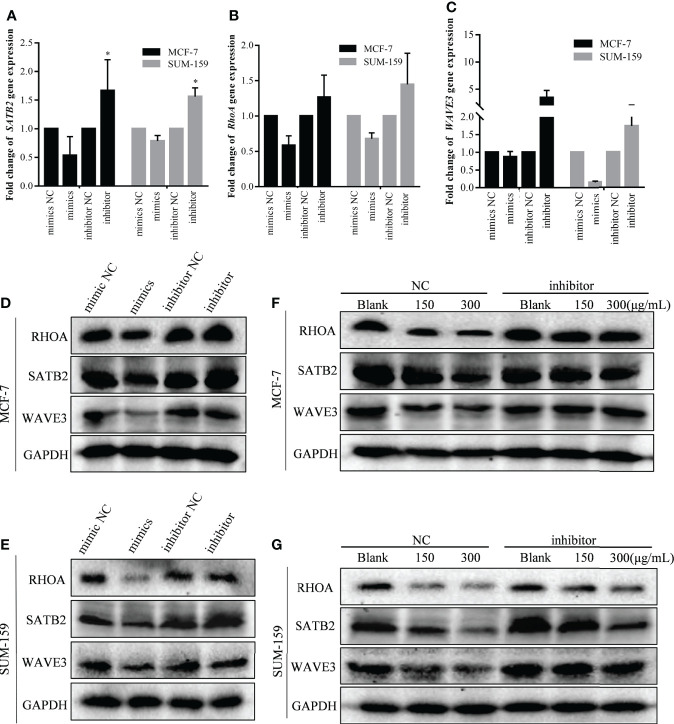
The effect of GBK treatment on miR-31 target gene expression after transfected with miR-31 mimics and inhibitor in MCF-7 and SUM-159 cells. **(A–C)** The MCF-7 and SUM-159 cells were transfected with miR-31 mimics or inhibitor, and different concentrations of GBK were added. After 24 hours, expression of *SATB2*, *RHOA* and *WAVE3* was analyzed by RT-qPCR. Data are presented as mean ± s.e.m. of three independent experiments. Student’s t test, * p<0.05. **(D, E)** Protein expression levels of miR-31 target genes after transfection with miR-31 mimics or inhibitor in MCF-7 and SUM-159 cells. Cell lysates were analyzed by western blot. **(F, G)** Protein expression levels of miR-31 target genes related to cell migration and invasion. Cells were transfected with miR-31 inhibitor for 24 h and then treated with GBK gradient for another 24 h in MCF-7 and SUM-159 cells. Cell lysates were analyzed by western blot. *GAPDH* was used as an internal control.

Western blot analysis was also performed to further validate the effect of GBK on the expression of invasion-metastatic related genes. It was demonstrated that the expression levels of *RHOA*, *WAVE3*, and *SATB2* were all reduced after GBK treatment in MCF-7 and SUM-159 cells (**Figures 3F, G** NC group). In order to demonstrate the direct involvement of miR-31 in the regulation of these genes in cancer cells, we tested the effect of miR-31 mimics and inhibitor on the expression of *STAB2*, *RHOA*, *WAVE3* and other relative genes after transfection into MCF-7 and SUM-159 cells (**Figures 3A, C**; **Supplementary Figure 5**). We found that miR-31 mimcs downregulated *STAB2*, *RHOA*, *WAVE3* expression, by contrast, miR-31 inhibitor upregulated *STAB2*, *RHOA*, *WAVE3* expression in both MCF-7 and SUM-159 cells (**Figures 3A–E**). Moreover, inhibition of miR-31 prevented the degradation of these proteins, orchestrated by GBK gradient (**Figures 3F, G** inhibitor group).

Thus, we can speculate that GBK inhibits the migration and invasion of breast cancer cells by promoting the expression of miR-31, which in turn impaires the expression of miR-31 target genes, such as *RHOA*, *WAVE3*, and *SATB2.*

### Expression of miR-31 and its host gene lncRNA LOC554202 is upregulated by inhibiting promoter hypermethylation after GBK treatment

**Figure 4:**
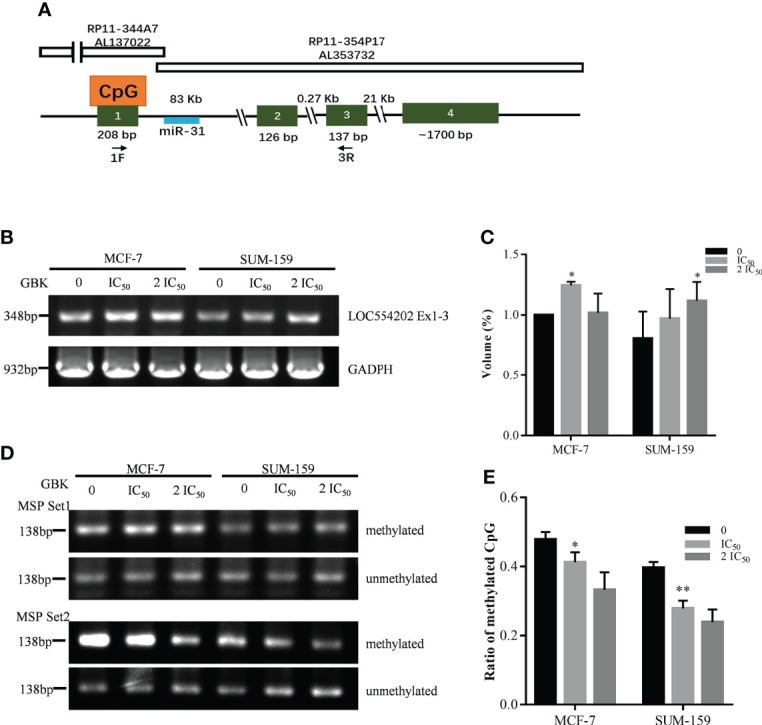
Gene expression and promoter hypermethylation of *LOC554202* are modified after GBK treatment in MCF-7 and SUM-159 cells. **(A)** Schematic representation of genomic organization of *LOC554202* gene [adapted from (27)]. **(B)** Semi-quantitative RT-PCR of *LOC554202* transcript in MCF-7 and SUM-159 cells. *GAPDH* was used as an internal control. Quantitative analysis of the data was shown in **(C)**. **(D)** Hypermethylation of two CpG sites in *LOC554202* promoter was detected by Methylation specific PCR (MSP) in MCF-7 and SUM-159 cells. Bisulfite-modified DNA extracted from the indicated samples was detected using 2 sets of primers. **(E)** Methylation ratios for all CpGs (including methylated and unmethylated) obtained by sequencing the bisulfite-modified DNA using Set2 primers. Data are presented as mean ± s.e.m. of three independent experiments. Student’s t test, *p < 0.05, **p < 0.01.

It has been documented that miR-31 is located in the intronic sequence of long non-coding RNA (lncRNA) *LOC554202*, and its transcriptional activity is regulated by *LOC554202* (**Figure 4A**) (27). It was also demonstrated that the major mechanisms for silencing miR-31 in TNBC is hypermethylation of the CpG island of the *LOC554202* promoter region, which may become a new entry point for TNBC treatment (27).

We tested the expression of *LOC554202* in the MCF-7 and SUM-159 cell lines and found that the expression of *LOC554202* was upregulated after GBK treatment (**Figures 4B, C**). Next, we selected two CpG sites within *LOC554202*, and methylation-specific PCR (MSP) technology was used to detect changes in the methylation level of these sites after GBK treatment (**Supplementary Figure 6**). We found that the methylation levels of CpG MSP Set2 decreased significantly under GBK treatment in a dose-dependent manner in the two cell lines (**Figures 4D, E**). These data indicate that GBK treatment affects the epigenetic modification at the CpG sites and plays an important role in the upregulation of both *LOC554202* and miR-31.

### Combination treatment with GBK and DDP/VP-16 has synergistic and dose reduction potential in the proliferation of breast cancer cells

**Figure 5:**
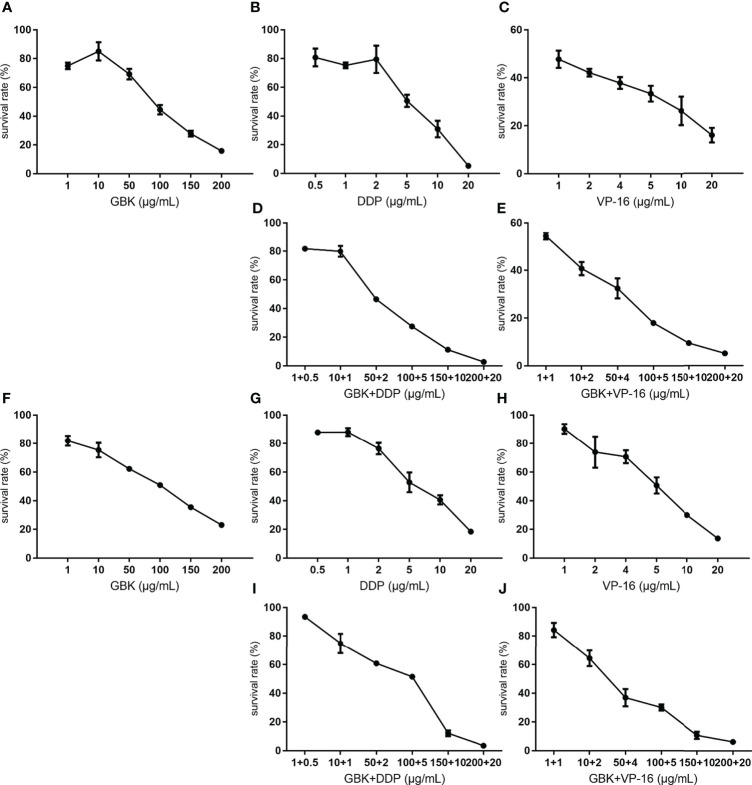
Combination treatment with GBK and DDP/VP-16 has synergistic and dose reduction potential in the proliferation of breast cancer cells. The growth inhibition assay was carried out using CCK-8 Kit, and the cell survival rate was measured 48 hours after drug administration. The sensitivity of SUM-159 and MCF-7 cell lines to growth inhibition 48 hours after exposure to GBK and DDP/VP-16 (IC50 concentrations for DDP/VP-16 was 5μg/ml) was determined. The survival rate of SUM-159 cells under different concentrations of **(A)** GBK, **(B)** DDP, **(C)** VP-16, **(D)** DDP and GBK, **(E)** VP-16 and GBK is showed in the top panel. The survival rate of MCF-7 cells under different concentrations of **(F)** GBK, **(G)** DDP, **(H)** VP-16, **(I)** DDP and GBK, **(J)** VP-16 and GBK is showed in the bottom panel. Data are presented as mean ± s.e.m. of three independent experiments.

The chemotherapy drugs cisplatin (DDP) and Vepeside (VP-16) are widely used as first-line therapy for metastatic breast cancer and small cell lung cancer 29, 30). In our previous study, we demonstrated that GBK exerted specific inhibitory effects on breast cancer cells but not on normal breast cells or cancer cell types (31). In this preclinical study, we want to evaluate the effect of GBK when used in combination with DDP/VP-16 to treat breast cancer cells. The sensitivity of SUM-159 and MCF-7 cell lines to growth inhibition was determined after a 48 hour incubation with GBK and DDP/VP-16, which was used as a single agent, or in combination at six different concentrations between 0.1× and 4× their respective IC50 for DDP, as well as 0.2× and 4× their respective IC50 for VP-16. The effect of the combined treatment on cell growth inhibition was cell type dependent. The combination of DDP/VP-16 with GBK at all concentrations led to greater growth inhibition compared to either agent alone in both SUM-159 (**Figures 5A–E**) and MCF-7 (**Figures 5F–J**) cell lines, although the increase was smaller for MCF-7 cells.

These results demonstrated that chemotherapy drugs DDP/VP-16 combination with GBK has synergistic and dose reduction potential in the proliferation of breast cancer cells SUM-159 and MCF-7, indicating a potential guiding significance for clinical combination treatment.

### Combination treatment with GBK and DDP impaired the independent viability of breast cancer cells

**Figure 6:**
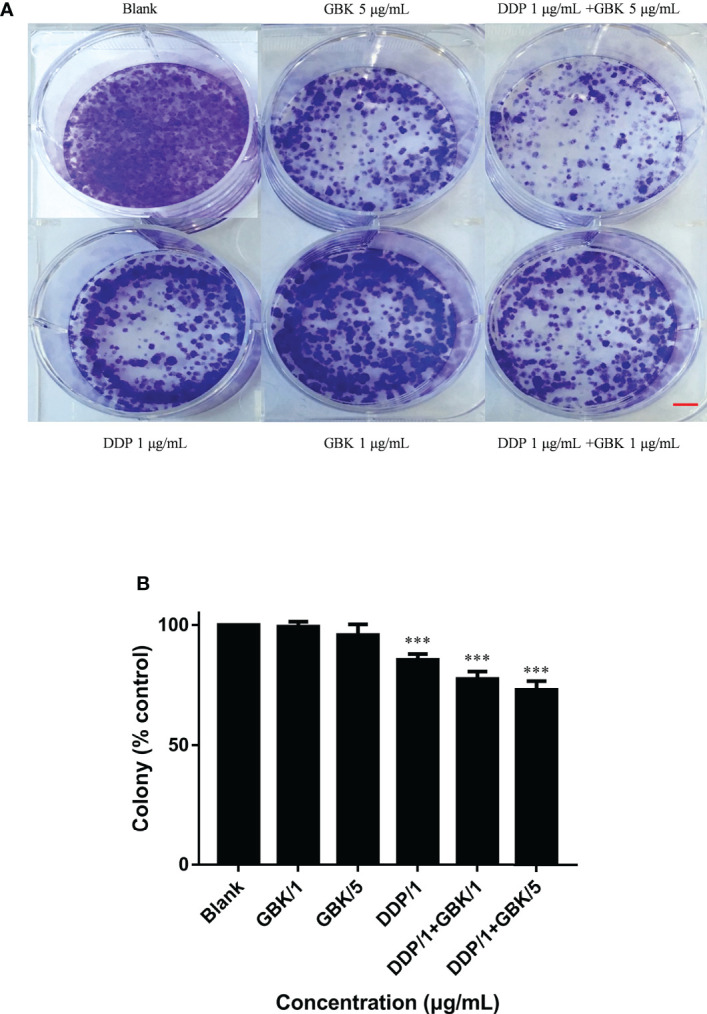
Combination treatment with GBK and DDP impairs the independent viability of breast cancer cells. The effect of combination treatment on the colony formation of breast cancer cells was analyzed. **(A)** Cell viability was determined by crystal violet staining of SUM-159 cells. **(B)** Quantification of the results in **(A)**. Data are presented as mean ± s.e.m. of three independent experiments. Student’s t test, *** p<0.0001. Scale bar represents 2.5 mm.

In colony formation experiments, the colony forming rate represents independent cell survival. The representative images after crystal violet staining showed that GBK can inhibit the formation of cell colonies in TNBC SUM-159. Moreover, this effect is further enhanced when GBK is used in combination with DDP, and this inhibitory ability is positively correlated with drug concentration (**Figure 6A**). The quantitative data also indicated that a combination of GBK and DDP treatment had a synergistic inhibitory effect on breast cancer cell colony formation (**Figure 6B**).

The combination treatment with DDP and GBK has a more obvious and stable inhibitory effect on the colony formation than that in VP-16 and GBK treatment group(data not shown), therefore DDP and GBK strategy was chosen to conduct tumor xenograft model experiments in nude mice.

### The effect of GBK and DDP combination treatment on miR-31 target gene expression

**Figure 7:**
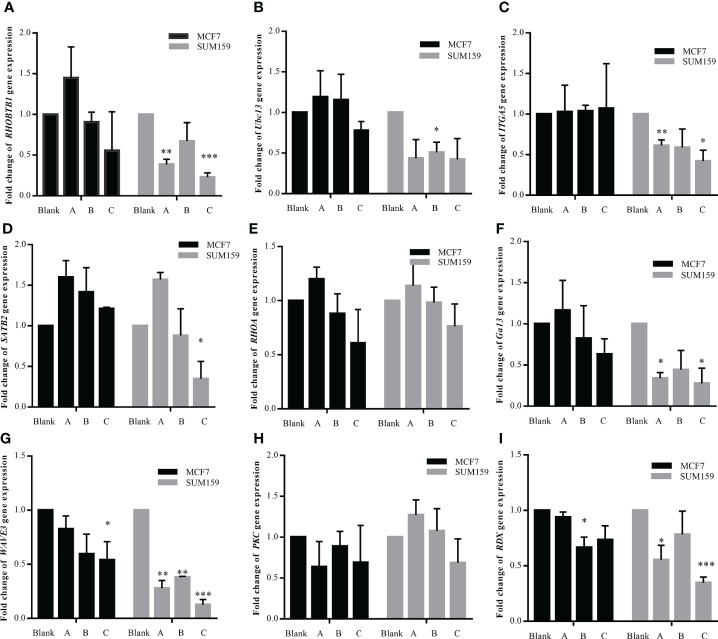
**(A–I)** Effect of combination treatment with GBK and DDP on miR-31 target gene expression. The effect of combination treatment with GBK and DDP on the expression of miR-31 target genes was detected by RT-qPCR. Three experimental groups were established, 1 μg/ml DDP (group A), 1 μg/ml DDP and 1 μg/ml GBK (group B), and 1 μg/ml DDP and 5 μg/ml GBK (group C). Double distilled H_2_O was added to the control group (Blank). Black bars indicate MCF-7 cells and gray bars indicate SUM-159 cells. Data are presented as mean ± s.e.m. of three independent experiments. Student’s t test, *p < 0.01, **p < 0.001, ***p < 0.0001.

It has been shown that GBK treatment causes the upregulation of miR-31 and its host gene lncRNA *LOC554202* in breast cancer cells when acting alone. In order to study whether the combination of GBK and DDP could also have the same synergistic function, RT-qPCR was performed to detect the expression of miR-31-targeted genes in SUM-159 and MCF-7 cells after drug treatment. It was revealed that when SUM-19 cells were treated with DDP and high dose of GBK combination, the expression of *RHOBTB1* (**Figure 7A**), *ITGA5* (**Figure 7C**), *SATB2* (**Figure 7D**), *WAVE3* (**Figure 7G**) and RDX (**Figure 7I**) decreased more significantly than those using DDP alone. Meanwhile, changes in the expression of other genes were not significant (**Figures 7B, E, F, H**). The synergistic effect of GBK and DDP combination therapy was more pronounced in SUM-159 cells then that in MCF-7 cells, indicating that the combination treatment has better effect on inhibition of gene expression in the more aggressive breast cancer cell line.

### Combination treatment with GBK and DDP has a synergistic and dose dependent effect on the inhibition of tumor growth *in vivo*

To further evaluate whether the combination treatment with GBK and DDP had a clear inhibitory effect on breast cancer progression *in vivo*, NOD/SCID immunodeficient mice were used to construct a human breast cancer cell xenograft model by subcutaneously inoculating mice with SUM-159 cells. All of the mice developed subcutaneous xenografts, prior to being divided into four groups and treated intraperitoneally with GBK 10 mg/(kg.1 Day) and DDP 1 mg/(kg.2 Days) in group A, GBK 5 mg/(kg.1 Day) and DDP 1 mg/(kg.2 Days) in group B, 0.9% saline in group C, and DDP 1 mg/(kg.2 Days) in group D, when the tumor volume reached 8 mm^3^. Mice were monitored for the next 24 days of continuous injection, and the notable anti-tumor effects were observed.

**Figure 8:**
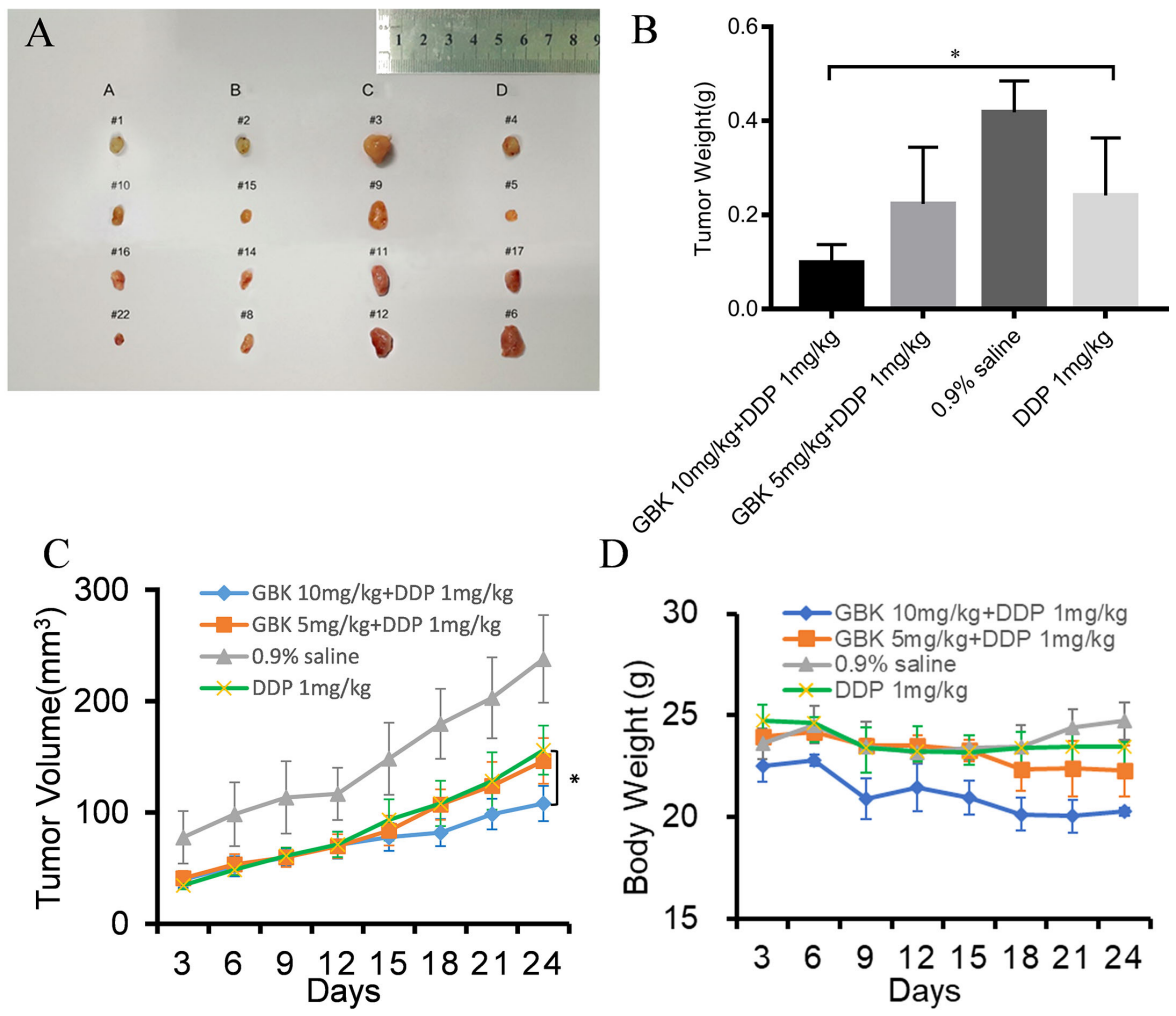
The anti-tumor effect of combination treatment with GBK and DDP on breast cancer. **(A)** SUM-159 cells were injected into the flanks of NOD/SCID mice, and when the tumors had grown to ~5–6 mm in diameter, the following treatment regiments were administered intraperitoneally as described for 24 days: GBK 10 mg/(kg.1D) and DDP 1 mg/(kg.2Ds) in group A, GBK 5 mg/(kg.1D) and DDP 1 mg/(kg.2Ds) in group B, 0.9% saline in group C, and DDP 1 mg/(kg.2Ds) in group D. **(B)** On day 24, tumors were excised and subjected to weight analysis. **(C)** On day 0, the tumor size was normalized to 1 for all the groups. Tumor volume was monitored and measured every 3 days. **(D)** Body weights of the NOD/SCID mice in groups A, B, C, and D were monitored for 24 days. Data are presented as mean ± s.e.m. of four independent mice. Student’s t test, *p < 0.01.

We found that DDP alone (group D) could inhibit tumor proliferation in the xenograft model. The combined treatment of GBK and DDP resulted in a further reduction in the tumor volume and the average tumor weight of mice in groups A and B, in comparison to group D. In day 24, we observed a significant decrease in the tumor weight and tumor volume in group A compared with group D (**Figures 8B, C**), indicating that DDP and GBK worked synergistically in a dose dependent manner in inhibiting breast cancer cell proliferation *in vivo* (**Figures 8A–C**).

In addition, the body weights of mice in group C (which were given saline) increased slightly, while the body weights of mice in group D (receiving DDP alone) decreased slightly. Interestingly, the body weights of mice in groups A and B (receiving GBK and DPP in combination) decreased markedly, with group A mice experiencing more pronounced weight loss compared to group B (**Figure 8D**). It was indicated that GBK could also perform as an antihyperlipidemic drug alone with its anti-tumor properties.

## Discussion

In this study, we investigated the mechanisms underlying the GBK-mediated impairment of breast cancer cell invasion and metastasis at the cellular and molecular levels. GBK exerts a significant inhibitory effect on the proliferation and migration of breast cancer cells, in a dose dependent manner. We also identified the main target of GBK by analyzing breast cancer-associated miRNA expression pattern changes during GBK treatment. We found that the expression of both miR-31 and its host gene *LOC554202* was upregulated following GBK treatment. Meanwhile, the miR-31 target genes, associated with cell migration and metastasis, were downregulated, indicating that GBK may inhibit cell migration by promoting the expression of tumor suppressor miR-31. Moreover, we demonstrated that the actions of GBK in inhibiting the growth and migration of TNBC cells were further enhanced when used in combination with the chemotherapy drug DDP, both *in vitro* and *in vivo*.

Given the large number of studies reporting the link between abnormal miRNA expression and a number of human diseases, it is evident that these molecules are key regulators of many biological processes. In addition, miRNAs play role in the regulation of cancer growth. While some miRNAs can be used as prognostic markers of malignant tumors, others are potential targets for cancer treatment 32). Considering that the expression of miRNAs affects almost every stage of malignant tumor formation (occurrence, development, metastasis, and drug resistance) (33) and that it is relatively stable and can be detected in a variety of biological fluids (such as blood, urine, cerebrospinal fluid and saliva) (34), miRNAs may become valuable biomarkers in cancer therapy.

**Figure 9:**
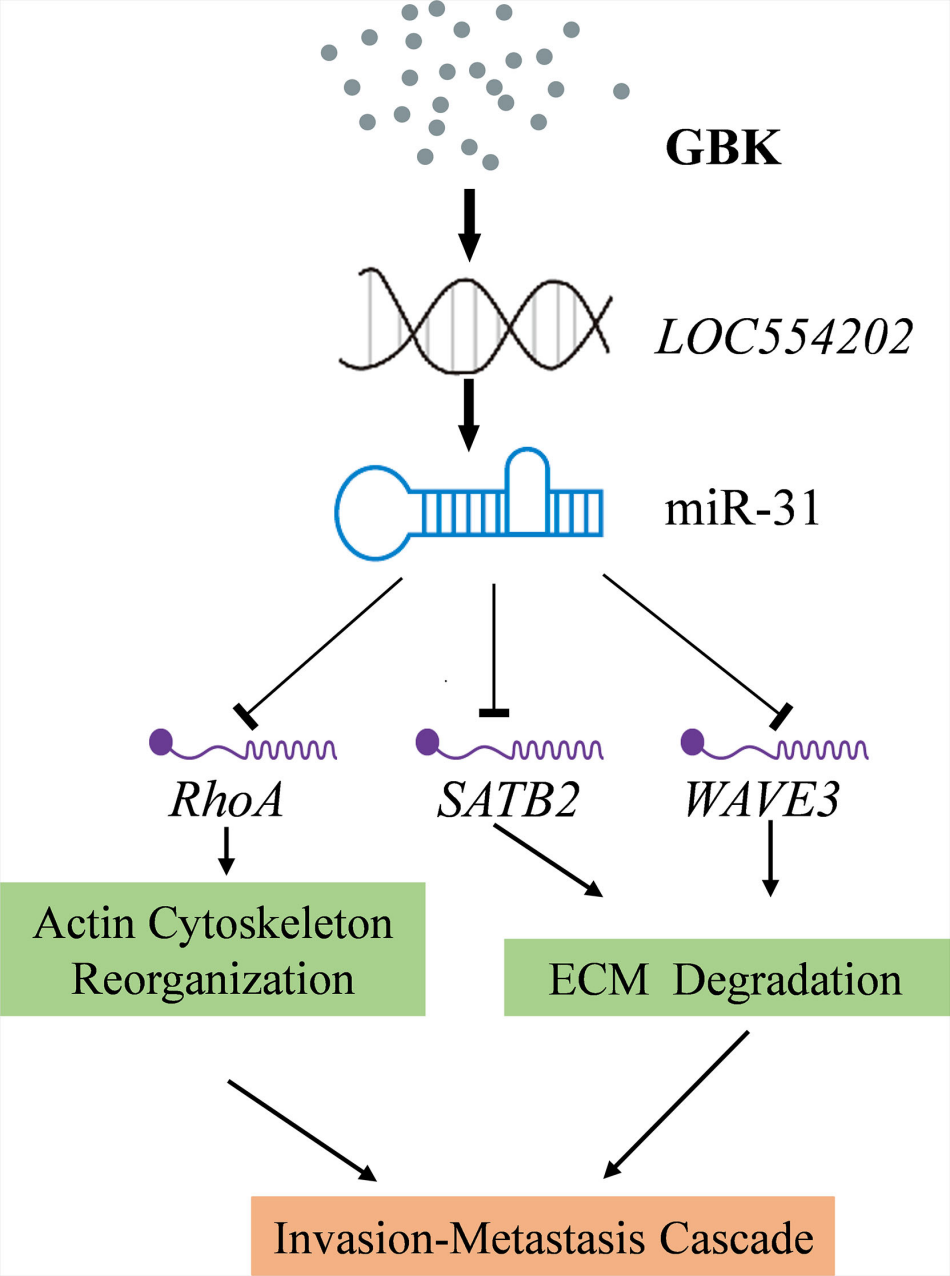
A proposed signaling network for GBK in inhibiting the migration and invasion of breast cancer cells. GBK treatment affects the epigenetic modification at CpG sites by downregulating DNA methyltransferases. Thus, the methylation levels at CpG of lncRNA *LOC554202* decreased significantly following GBK treatment in a dose-dependent manner in breast cancer cell lines, upregulating both *LOC554202* and miR-31. miR-31 directly acts on the 3’UTR region of multiple target genes, such as *RHOA*, *SATB2*, and *WAVE3*, which playing roles in cancer cell invasion, migration, and proliferation. GBK treatment impairs the expression of RhoA, SATB2, and WAVE3.

One especially interesting cancer-related miRNA is miR-31, which is frequently altered in a large variety of cancers (35, 36). For example, in breast cancer, the loss of miR-31 expression is associated with a high risk of metastases (35). Existing studies have demonstrated that miR-31 is a tumor suppressor with an important role in the occurrence and development of cancer. miR-31 directly acts on the 3’UTR region of multiple target genes such as *RHOA*, *SATB2*, or *WAVE3*, which are implicated in cell invasion, migration, and proliferation. RhoA is required for the motility and migration of breast cancer cells, through its involvement in actin and microfilament skeleton polymerization (37). The RhoA/Rho-associated coiled coil-forming protein kinase (ROCK) signaling pathway plays an important role in this process. In addition to directly affecting cell microfilament skeleton polymerization, it also affects the degradation of the extracellular matrix (ECM) (38), while activated ROCK can promote tumor cell invasion and metastasis 39). Special AT-rich sequence-binding protein-2 (SATB2) is an important nuclear matrix protein that participates in actin cytoskeleton regulation (40). The main targets of SATB2 are matrix metalloproteinase-3 (MMP3) and TIMP3 (41). The extracellular domain of cell adhesion factor E-cadherin can be cleaved by MMP3 directly, which facilitates the metastasis of cancer cells (42). SATB2 also acts on the MEK5/ERK5 signaling pathway (43). Extracellular signal-regulated kinase 5 (ERK5) is a mitogen-activated protein kinase that can induce actin cytoskeleton remodeling and promote cell migration. WAVE3 has multiple downstream effectors. It was found that WAVE3 has an intricate regulatory relationship with the Akt-associated and NF-κB signaling pathways. The NF-κB signaling pathway is mainly involved in the degradation of the ECM (44). Based on existing experimental data, we postulated that the targets of miR-31 mentioned above act as potential downstream effectors of GBK. Thus, we propose a possible signaling network whereby GBK acts by inhibiting the migration and invasion of breast cancer cells (**Figure 9**). In this study, we have shown that GBK affects the expression of *RHOA*, *SATB2*, and *WAVE3*. Whether the target proteins and signaling pathways regulated by these three genes are also affected by GBK needs to be further studied.

miR-31 is located in the intronic sequence of lncRNA *LOC554202*, and its transcriptional activity is regulated by *LOC554202*. It has been documented that the major mechanism responsible for silencing miR-31 in TNBC is the hypermethylation of the CpG island in the *LOC554202* promoter region (27). We demonstrated previously that the transcription of DNA methyltransferase *DNMT1*, *DNMT3A*, *DNMT3B*, and *COQ3* genes were downregulated in GBK-treated MCF-7 cells (31). In this study, we found that the methylation levels at the CpG of lncRNA *LOC554202* decreased significantly under GBK treatment in a dose-dependent manner in breast cancer cell lines (45). Therefore, GBK treatment affects the epigenetic modification at the CpG sites by downregulating DNA methyltransferases and plays an important role in the upregulation of both the *LOC554202* and miR-31 (**Figure 9**).

In addition, miRNAs are known to affect many cellular processes *via* their ability to post-transcriptionally control gene expression. It is therefore important to identify other miRNA target genes, examine how miRNAs are regulated, and study their involvement in cellular functions. High-throughput bioinformatics analysis could offer a better method for studying the mechanism of action of GBK, and also benefit clinical applications.

DDP is an alkylating agent classified as an anti-neoplastic drug that has been extensively used in the treatment of advanced breast cancer, especially in metastatic breast cancer and TNBC. However, several adverse side effects limit its long-term usage. Combination treatment with other anti-tumor agents is an effective way to solve this problem. The combination of GBK and DDP treatments appear to work synergistically to inhibit the proliferation of breast cancer cells (especially in TNBC) *in vitro* and *in vivo*, and could be used to potentially reduce the individual doses of these drugs required to achieve the same effect in the clinic. Moreover, the expression of the miR-31 target genes *SATB2* and *RHOA* was significantly reduced in SUM-159 cells after combination treatment with GBK and DDP, indicating that GBK could impair breast cancer cell migration and invasion.

## Conclusions

In conclusion, in this study we identified the target of GBK and explored the underlying mechanisms involved in its inhibition of breast cancer migration and invasion, especially in TNBC, thus providing theoretical support for the development of GBK as an auxiliary drug in the clinical treatment.

## Data availability statement

The original contributions presented in the study are included in the article/**Supplementary Material**. Further inquiries can be directed to the corresponding author.

## Ethics statement 

The animal study was reviewed and approved by Institutional Animal Care and Use Committee at Inner Mongolia University.

## Author contributions

Data curation, LF and MMo; funding acquisition, LF; investigation, XT, JL, KN, LZ, and YL; methodology, MMa. All authors contributed to the article and approved the submitted version.

## Funding

This work was supported by grants from the National Natural Science Foundation of China (NSFC Grant no. 31960162 to LF), and the Program for Young Talents of Science and Technology in Universities of Inner Mongolia Autonomous Region (Grant no. NJYT-17-B03 to LF),

## Acknowledgments

GBK is a generous gift from Professor Gereltu Borjihan of Inner Mongolia University.

## Conflict of interest

The authors declare that the research was conducted in the absence of any commercial or financial relationships that could be construed as a potential conflict of interest.

## Publisher’s note

All claims expressed in this article are solely those of the authors and do not necessarily represent those of their affiliated organizations, or those of the publisher, the editors and the reviewers. Any product that may be evaluated in this article, or claim that may be made by its manufacturer, is not guaranteed or endorsed by the publisher.

## Abbreviations

miRNAs, MicroRNAs; GBK, potassium piperate; GBN, piperlonguminine; GBO, piperine; ncRNAs, non-coding RNA; lncRNA: long non-coding RNA; CLL, chronic lymphocytic leukemia; DNMT1, DNA methyltransferases-1; ROS, reactive oxygen species; RT-qPCR, real-time quantitative polymerase chain reaction; TNBC: triple-negative breast cancer; MSP: methylation-specific PCR; ELISA: Enzyme linked immunosorbent assay; MMP3: matrix metalloproteinase-3; ECM: extracellular matrix; ERK5: Extracellular signal-regulated kinase 5; DDP, Cisplatin; VP-16, etoposide; ROCK, rho associated coiled coil-forming protein kinase; PBS, hosphate buffered solution; CCK-8, Cell Counting Kit-8; PFA, Paraformaldehyde;MSP, Methylation specific PCR; SATB2, special AT-rich sequence-binding protein-2.
