# Supplementary material for: The antihyperlipidemic drug potassium piperonate impairs the migration and tumorigenesis of breast cancer cells via the upregulation of miR-31
Source: Front Oncol. 2022 Oct 13;12:828160. doi: 10.3389/fonc.2022.828160 (PMC9606244; doi:10.3389/fonc.2022.828160)
Supplement: Supplementary file 1 [file DataSheet_1.docx]

**Supplementary Figures**


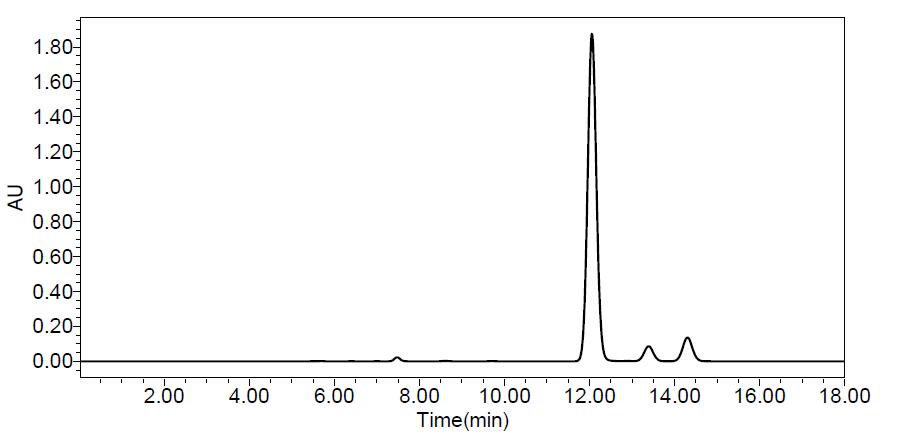


**Supplementary Figure 1. High-pressure liquid chromatography to verify GBK purity**

**
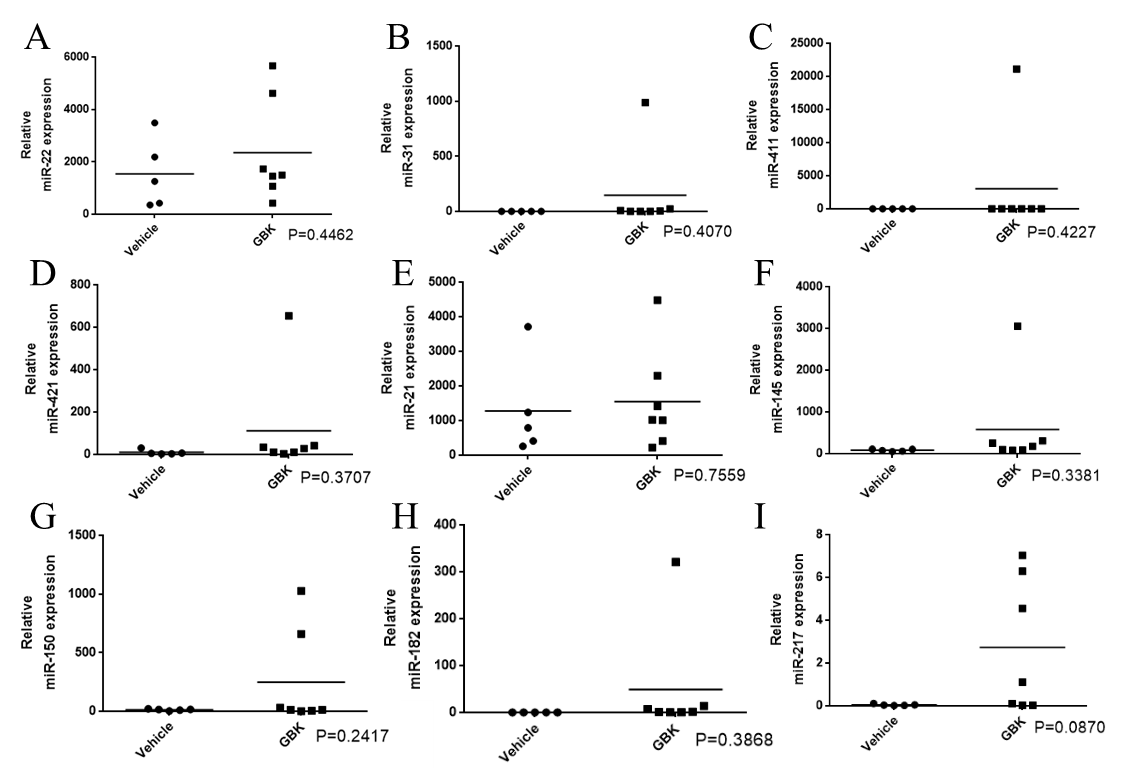
**

**Supplementary Figure 2. Validation of miRNAs expression levels in beast cancer xenograft mouse serum after GBK treatment by RT-qPCR analysis**

The mice in the experimental group were administered with GBK, and other feeding conditions were consistent with the control group. Serum samples were collected and miRNA expression was detected by RT-qPCR. Expression of (A) miR-22, (B) miR-31, (C) miR-411, (D) miR-421, (E) miR-21, (F) miR-145, (G) miR-150, (H) miR-182, and (I) miR-217 was detected by RT-qPCR. Student's t test, * p<0.05, ** p<0.01, *** p<0.001.


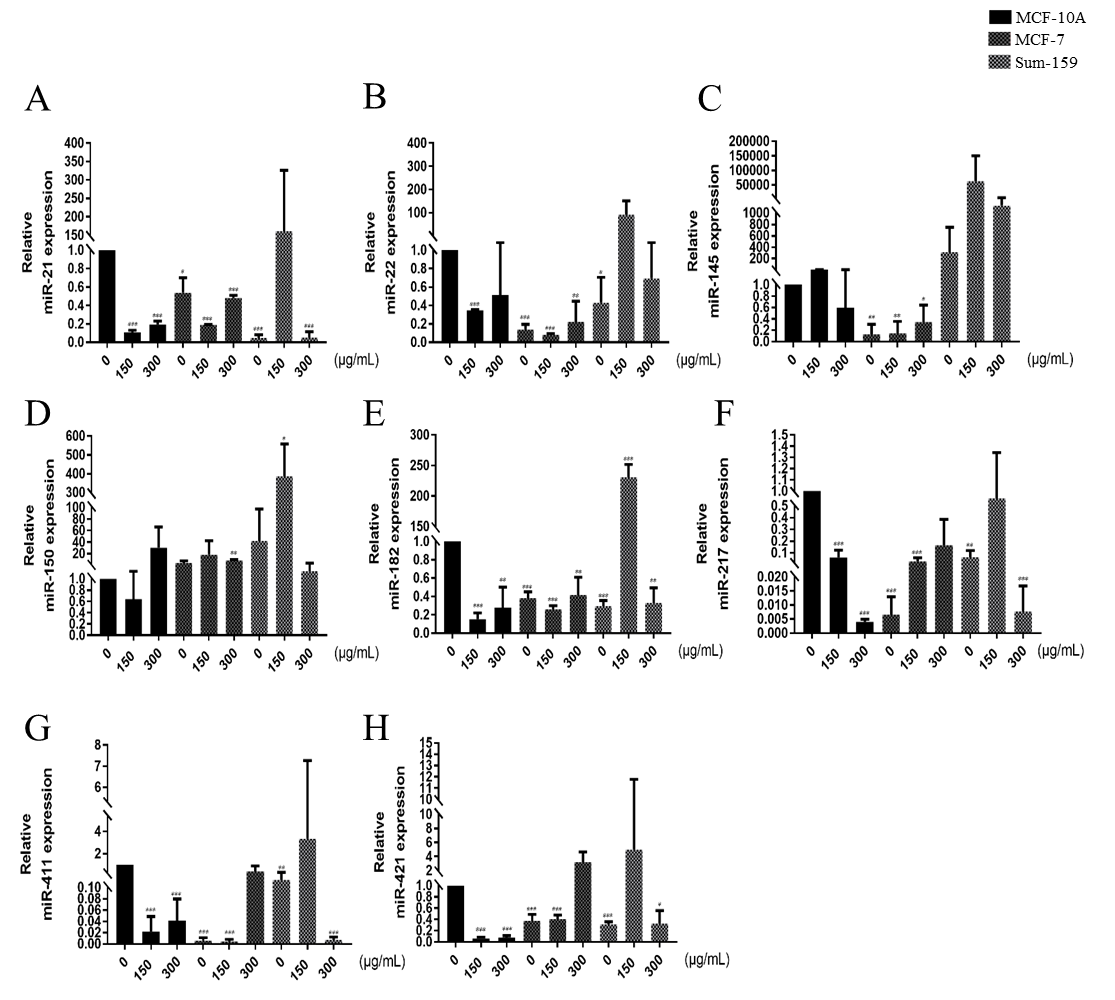


**Supplementary Figure 3. The expression pattern of eight miRNAs after GBK treatment in three different breast cell lines**

MCF-10A, MCF-7, and SUM-159 cells were treated with different concentration of GBK. Total RNA was extracted after 48 hours for miRNA expression detection by RT-qPCR. (A-I) Quantification of miR-21, miR-22, miR-145, miR-150, miR-182, miR-217, miR-411 and miR-421 expression in three different breast cell lines after application of different concentrations of GBK. Data are presented as mean ± s.e.m. of three independent experiments. Student's t test, * p<0.05, ** p<0.01, *** p<0.001.


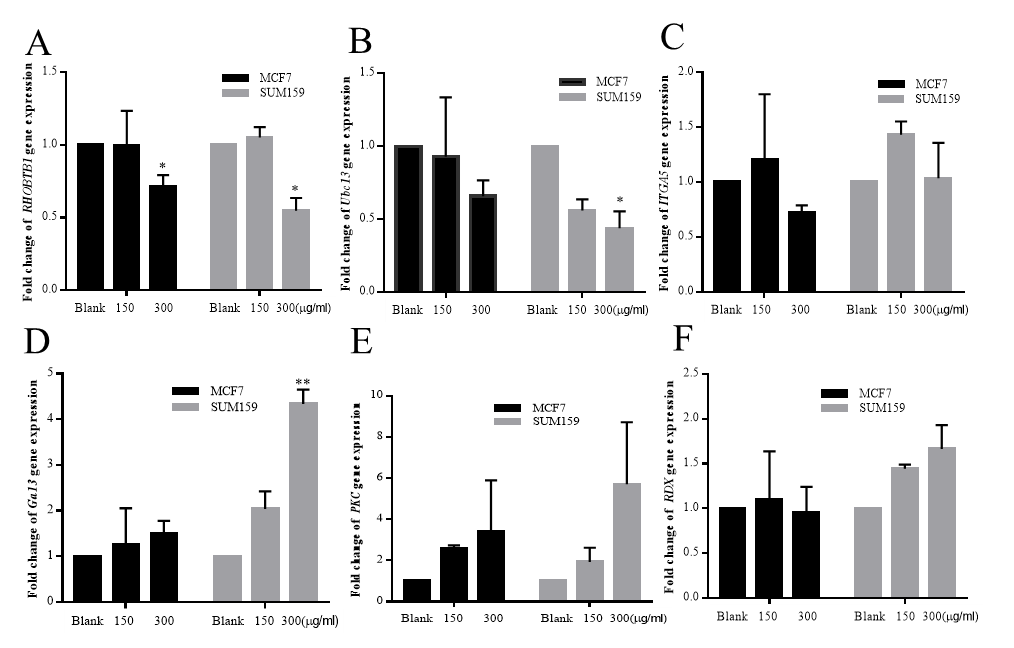


**Supplementary Figure 4. Detection of miR-31 target gene expression under the treatment of GBK in MCF-7 and SUM-159 cells.**

(A-F) Expression of *RHOBTB1, UBC13, ITGA5, Gα13, PKC,* and *RDX* in MCF-7 and SUM-159 cells after treatment with different concentrations of GBK was analyzed by RT-qPCR. Data are presented as mean ± s.e.m. of three independent experiments. Student's t test, * p<0.05, ** p<0.01, *** p<0.001.


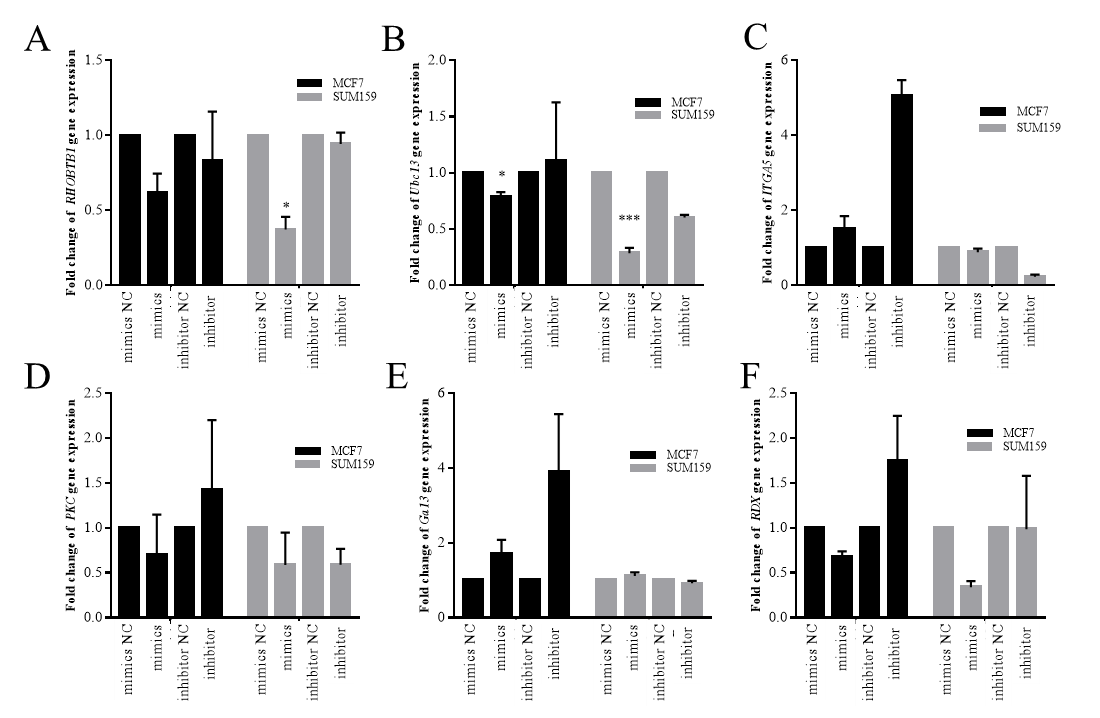


**Supplementary Figure 5. The differential expression of miR-31 target genes in MCF-7 and SUM-159 cells after transfected with miR-31 mimics and inhibitor.** The MCF-7 and SUM-159 cells were transfected with a miR-31 mimics or inhibitor. After 24 hours, expression of *RHOBTB1, UBC13, ITGA5, Gα13, PKC,* and *RDX* was analyzed by RT-qPCR. Data are presented as mean ± s.e.m. of three independent experiments. Student's t test, * p<0.05, ** p<0.01, *** p<0.001.


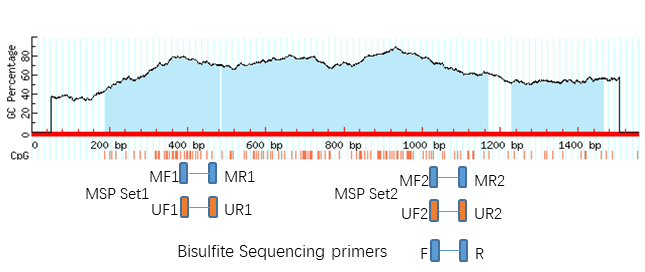


**Supplementary Figure 6. The locations of the bisulfite sequencing primers**

Hypermethylation of two CpG sites in *LOC554202* promoter was detected by Methylation specific PCR (MSP) in MCF-7 and SUM-159 cells. Bisulfite-modified DNA extracted from the indicated samples was detected using 2 sets of primers.The locations of the bisulfite sequencing primers was adapted from（Augoff et al., 2012）.

**Supplementary tables**

**Supplementary table 1. miRNAs Primer list Ⅰ**

| Primer Name | Primer sequence (5'-3') |
| --- | --- |
| mmu-miR-21a | CCGTAGCTTATCAGACTGATGTTG |
| mmu-miR-22 | GCGAAGCTGCCAGTTGAAGAACT |
| mmu-miR-31 | GAGGCAAGATGCTGGCATAGCTGAA |
| mmu-miR-145a | CGTCCAGTTTTCCCAGGAATCCC |
| mmu-miR-150 | GTCTCCCAACCCTTGTACCAGTG |
| mmu-miR-182 | GCTTTGGCAATGGTAGAACTCACAC |
| mmu-miR-217 | CGTACTGCATCAGGAACTGACTGGA |
| mmu-miR-411 | CGTAGTAGACCGTATAGCGTA |
| mmu-miR-421 | GCATCAACAGACATTAATTGGGCG |
| has-mir-21 | CCGTAGCTTATCAGACTGATGTTG |
| has-mir-22 | GCGAAGCTGCCAGTTGAAGAACT |
| has-mir-31 | GAGGCAAGATGCTGGCATAGCTGAA |
| has-mir-145 | CGTCCAGTTTTCCCAGGAATCCC |
| has-mir-150 | GTCTCCCAACCCTTGTACCAGTG |
| has-mir-182 | GCTTTGGCAATGGTAGAACTCACAC |
| has-mir-217 | CGTACTGCATCAGGAACTGACTGGA |
| has-mir-411 | CGTAGTAGACCGTATAGCGTA |
| has-mir-421 | GCATCAACAGACATTAATTGGGCG |

*Note: the internal reference gene miR- 39 (miRNeasy® Serum/ Plasma Spike-In Control) and the 3' microRNA Primer universal downstream primer (Mir-XTM miRNA First-Strand Synthesis Kit) are provided by the corresponding kit.*

**Supplementary table 2. Primer list Ⅱ**

| Gene name | Forward primer sequence | Reverse primer sequence |
| --- | --- | --- |
| *RhoA* | GGACTTAAGCGTCTGGCTC | AGTGCCACCCATGAGAACTG |
| *WAVE3* | CCGCGGACCGTTTTAGTT | GACGCTCCATGGTACACACT |
| *Gα13* | AGGGAACTTTTTGCCCGAGA | ACCCTCATACCTGACCGTGA |
| *Ubc13* | AGGCCTTGTTAAGTGCTCCC | GCAAAATCCACCTCTTGCCAT |
| *ITGA5* | GGCTTCAACTTAGACGCGGA | GGCCGGTAAAACTCCACTGA |
| *RDX* | ACAGTTGGTTTGCGTGAGGT | TGTTCCAATACACGCTGGGG |
| *SATB2* | ATCGGAAACCGAGGACAACC | GTGTCTTCTTCTGGTGCGGA |
| *PKC* | CCATGTCGCCATTTCTTCGG | GCCCGTTCTCTGATTCGACA |
| *RHOBTB1* | CGGCTTCAGGGTAAGTCCAG | CCTGCCATATGCAAAGCGTC |
| *β-actin* | TTAGTTGCGTTACACCCTTTC | ACCTTCACCGTTCCAGTTT |

**Supplementary table 3. Primer list III**

| Gene name | Forward primer sequence | Reverse primer sequence |
| --- | --- | --- |
| *GAPDH* | TGAAGGTCGGAGTCAACGATTTGGT | CATGTGGGCCATGAGGTCCACCAC |
| *LOC554202 Ex1* | CAGAGCTGGGAGGCGGTGTTC | - |
| *OC554202 Ex4* | - | CTCTACACTGGCCTTGAGGAGGTA |

**Supplementary table 4. miR-31 mimics and inhibitor**

| Oligo name | Forward primer sequence | Reverse primer sequence |
| --- | --- | --- |
| hsa-miR-31-5p-mimics | AGGCAAGAUGCUGGCAUAGCU | AGCUAUGCCAGCAUCUUGCCU |
| hsa-miR-31-5p inhibitor | - | AGCUAUGCCAGCAUCUUGCCU |
